# Isolation and Characterization of a Double Stranded DNA Megavirus Infecting the Toxin-Producing Haptophyte *Prymnesium parvum*

**DOI:** 10.3390/v9030040

**Published:** 2017-03-09

**Authors:** Ben A. Wagstaff, Iulia C. Vladu, J. Elaine Barclay, Declan C. Schroeder, Gill Malin, Robert A. Field

**Affiliations:** 1 Department of Biological Chemistry, John Innes Centre, Norwich Research Park, Norwich NR4 7UH, UK; ben.wagstaff@live.co.uk (B.A.W.); vladu.iulia@yahoo.com (I.C.V.); elaine.barclay@jic.ac.uk (J.E.B.); 2 Marine Biological Association of the UK, Plymouth PL1 2PB, UK; dsch@mba.ac.uk; 3 Centre for Ocean and Atmospheric Studies, School of Environmental Sciences, University of East Anglia, Norwich Research Park, Norwich NR4 7TJ, UK; g.malin@uea.ac.uk

**Keywords:** *Prymnesium parvum*, haptophyte, algal bloom, algal virus, *Megaviridae*, NCLDV

## Abstract

*Prymnesium parvum* is a toxin-producing haptophyte that causes harmful algal blooms globally, leading to large-scale fish kills that have severe ecological and economic implications. For the model haptophyte, *Emiliania huxleyi*, it has been shown that large dsDNA viruses play an important role in regulating blooms and therefore biogeochemical cycling, but much less work has been done looking at viruses that infect *P. parvum*, or the role that these viruses may play in regulating harmful algal blooms. In this study, we report the isolation and characterization of a lytic nucleo-cytoplasmic large DNA virus (NCLDV) collected from the site of a harmful *P. parvum* bloom. In subsequent experiments, this virus was shown to infect cultures of *Prymnesium* sp. and showed phylogenetic similarity to the extended *Megaviridae* family of algal viruses.

## 1. Introduction

The last two decades have seen a boom in the study of marine viruses and the role that they play in regulating both bacterial and unicellular eukaryote bloom dynamics [1,2]. Although phages and the bacteria that they infect have been studied for many years, the more recently discovered *Acanthamoeba polyphaga mimivirus* (APMV) and its *Megaviridae* relatives have brought about a new age in photosynthetic protist virology. It has recently been shown that dsDNA viruses infecting algae do not form monophyletic lineages [3], with divergence occurring even within the host division. A good example of this evolutionary divergence can be found in viruses that infect the coccolithophore *Emiliania huxleyi* (EhV) [4,5] and the prymnesiophyte *Phaeocystis globosa* (PgV) [6], which along with other algal viruses have been proposed to form an extended branch of the *Megaviridae* [7]. It is widely accepted that these viruses not only play a crucial role in ecosystem dynamics [8,9], but also contribute significantly to biogeochemical cycles [10,11]. A lesser studied impact, however, lies in the role that such viruses may play in the termination of toxic eukaryotic algal blooms. Lytic viruses that infect the toxic raphidophyte *Heterosigma akashiwo* have been extensively studied [12,13,14,15,16,17,18,19] but, because of the elusive nature of *H. akashwio* toxicity to fish, none of these studies sought to investigate the role of viral infection on levels of algal toxicity.

The toxin-producing haptophyte *Prymnesium parvum* forms dense blooms in marine, brackish and inland waters, devastating fish populations through the release of natural product toxins [20,21]. The haptophytes are a diverse division of microalgae that include the bloom-forming *Emiliania huxleyi* and *Phaeocystis globosa*, both of which play crucial roles in oceanic carbon and sulfur cycles [22,23]. Virus infection of these organisms has been studied in some detail, with the genome of the dsDNA *Phaeocystis globosa* virus (PgV-16T) being recently described [3]. From a metabolomics perspective, *Phaeocystis pouchetti* lysis by a strain-specific virus has been shown to cause substantial release of dimethyl sulphide and its major precursor dimethylsulphoniopropionate [24], an action that is believed to contribute significantly to the global sulfur cycle. Although much effort has gone into studying the relationship between *E. huxleyi* and its infecting viruses, viruses infecting toxin-producing algal species within the haptophyte family are much less well studied. These include the euryhaline species *Prymnesium* spp. and *Chrysochromulina* spp., whose blooms can often result in severe economic damage through loss of fish stocks [25,26]. Viruses that infect the non-toxic *P. kappa* have recently been described, but to date no viruses have been isolated and characterized that infect the toxin-producing *P. parvum* species, even though Schwierzke et al. have previously suggested a role for viruses in regulating natural *P. parvum* populations [27].

In this study, we isolated a novel lytic virus of *P. parvum* 946/6, *Prymnesium parvum* DNA virus BW1 (henceforth referred to as PpDNAV), from the site of a recent harmful bloom event of this species in Norfolk, England. We show that the virus has a typical narrow host range; using morphological characterisation and phylogenetics, we also show that the virus lies in the recently described clade of algal megaviruses. 

## 2. Materials and Methods

### 2.1. Prymnesium parvum Culture Conditions

For choice of host cell, *P. parvum* 946/6 was obtained from the Culture Collection of Algae and Protozoa (CCAP—www.ccap.ac.uk). The additional 14 strains used for host range screening were obtained from the Marine Biological Association Culture Collection (https://www.mba.ac.uk/culture-collection/). Batch cultures were maintained at 22 °C on a 14:10 light cycle at 100 µmol·photons·m^−2^·s^−1^. Cultures were grown in f/2–Si medium at a salinity of 7–8 practical salinity unit (PSU). Under these conditions, cell densities of ~3 × 10^6^ cells·mL^−1^ could be achieved after 12–16 days of growth.

### 2.2. Isolation of Lytic Virus Particles

PpDNAV was isolated from surface water samples taken at various locations on Hickling Broad, Norfolk, England on 9 February 2016. In brief, 4 × 100 mL water samples from various locations around the Broad were centrifuged at 3000× *g* and the supernatant subsequently filtered through 0.45 µm pore-size filters (Sartorius AG, Goettingen, Germany). The resulting solutions were then concentrated 100- to 200-fold using 100 kDa MW cut off spin filters (Amicon Ultra 15, Merck Millipore, Watford, UK) to give 0.5 to 1 mL of viral concentrate, which was stored at 4 °C in the dark until use. Small volumes (0.2 mL) of concentrate from each location were added to 1.8 mL of exponentially growing cultures of *P. parvum* 946/6. Blank culture medium was used as a control. Cultures were visually inspected for signs of cell lysis (culture clearing) after 7–10 days where the control cultures continued to grow. Culture clearing was then followed up by Transmission Electron Microscopy (TEM) analysis of the culture lysates. Clonal populations of PpDNAV were obtained by taking the supernatant of a lysed culture, and exhaustively diluting with media. These diluted samples (0.2 mL) were added to 1.8 mL of an exponentially growing culture of *P. parvum* 946/6. The highest dilution that still produced cell lysis after seven days was taken through to the next round. This was repeated at least three times and resulted in a population of PpDNAV free of morphologically different viruses, as judged by TEM.

### 2.3. Transmission Electron Microscopy

For TEM analysis of virus-like particles (VLPs) in culture supernatant, 2 mL of a virus-lysed culture was filtered through 0.45 µm filters and 10 µL of the filtrate was adsorbed onto a 400 mesh copper palladium grid with a carbon-coated pyroxylin support film before being negatively stained with 2% aqueous uranyl acetate [28]. The grids were viewed in a FEI Tecnai 20 transmission electron microscope (Eindhoven, The Netherlands) at 200 kV and digital TIFF images were taken with an AMT XR60B digital camera (Deben, Bury St Edmunds, UK).

For analysing intracellular VLPs, 1 mL of infected cultures of *P. parvum* 946/6 was taken at 24 and 48 h post-infection (p.i.). These were centrifuged at 3000× *g* to pellet algal cells and the supernatant was discarded. The pellet was washed twice with sterile medium. The pelleted cells were then resuspended in 2.5% (*v*/*v*) aqueous glutaraldehyde solution and left overnight. This suspension was then centrifuged at 3000× *g* to pellet the algal cells. Half the volume of the supernatant was then discarded and an equal volume of warm (60 °C) low gelling temperature agarose (Sigma Aldrich, Haverhill, UK) was added, before resuspension of the cells and placing on ice to solidify. The solidified samples were then put into 2.5% (*v*/*v*) glutaraldehyde with 0.05 M sodium cacodylate, pH 7.3 [29] and left overnight. Using a Leica EM TP machine (Leica Microsystems, Cambridge, UK), the samples were washed in 0.05 M sodium cacodylate and then post-fixed with 1% (*w*/*v*) OsO4 in 0.05 M sodium cacodylate for 60 min at room temperature. After washing and dehydration with ethanol, the samples were gradually infiltrated with LR White resin (London Resin Company, London, UK) according to the manufacturer’s instructions. After polymerization, the resulting material was sectioned with a diamond knife using a Leica EM UC6 ultramicrotome (Leica Microsystems). Ultrathin sections of approximately 90 nm were picked up on 200 mesh gold grids that had been coated in pyroxylin and carbon. The grids were then contrast-stained with 2% (*w*/*v*) uranyl acetate for 1 h and 1% (*w*/*v*) lead citrate for 1 min, washed in distilled water and air-dried. The grids were then viewed with a FEI Tecnai 20 transmission electron microscope (Eindhoven, The Netherlands) at 200 kV and digital TIFF images were produced.

### 2.4. Host Specificity

Fifteen different strains of *Prymnesium* were tested in triplicate for signs of cell lysis by PpDNAV using the infection methodology described above. Cell lysis, as observed by culture clearing, was noted for five of the 15 strains tested (Table 1). 

### 2.5. Infection Cycle

The virus–algae lytic cycle was investigated by accurately recording algal cell abundance during an infection cycle. A late-log phase culture of *P. parvum* 946/6 was infected with PpDNAV (0.1% *v*/*v*) and triplicate 2 mL aliquots were taken at various time points post infection (p.i.). These were diluted with 0.2 µm filtered seawater prior to counting using a Multisizer 3 Analyser (Beckman Coulter, High Wycombe, UK) fitted with a 100 µm aperture tube. The control culture continued to grow throughout the experiment, whilst the infected algal culture was lysed rapidly after 48 h. 

### 2.6. Chloroform Sensitivity

To test the virus sensitivity to chloroform, an adaptation of the method of Martínez Martínez et al. was employed [30]. Briefly, 1 mL of 0.45 µm-filtered PpDNAV was added to an equivalent volume of chloroform and shaken vigorously for 5 min. The resulting mixture was then centrifuged at 4000× *g* in a benchtop centrifuge for 5 min to separate the organic and polar layers. The aqueous phase was transferred by pipetting to a clean microcentrifuge tube and incubated at 37 °C for 1 h to remove residual chloroform. As a control, 1 mL of chloroform was added to 1 mL of f/2 medium. Chloroform-treated PpDNAV, chloroform-treated medium and untreated PpDNAV were added to *P. parvum* 946/6 as described above in the infectivity experiment protocol; signs of lysis, as judged by culture clearing, were recorded after one week. 

### 2.7. Viral DNA Extraction, Sequencing, and Phylogenetic Analyses

For DNA extraction, 1 L of late log phase *P. parvum* 946/6 was infected with axenic PpDNAV (0.1% *v*/*v*). Lysis was allowed to occur over a period of five days, by which point almost all cells had been lysed. The culture was centrifuged at 6500× *g* to pellet cell debris, before being filtered through 0.22 µm filters to remove remaining cell debris or contaminating bacteria. The filtrate was incubated for 72 h with 100 μg/mL carbenicillin before being concentrated to 30 mL using 100 kDa mw cut-off spin filters. Ultracentrifugation at 150,000× *g* was used to pellet viral particles, and these were re-suspended in 2 mL of ρ = 1.4 CsCl and layered onto a CsCl gradient which was resolved at 150,000× *g* for 18 h. Fractions from ρ = 1.3 to ρ = 1.4 were pooled and DNA extracted using a PureLink Viral RNA/DNA Kit, according to the manufacturer’s protocol.

An amount of 1 µg of purified viral DNA was then sent to The Earlham Institute, UK, for Illumina MiSeq sequencing (Illumina, Inc., San Diego, CA, USA) and assembly. The initial assembly was then analysed using GeneMarkS [31] which identified 332 protein-coding sequences. BLASTp analysis was then performed against the National Center for Biotechnology Information (NCBI) GenBank nonredundant (nr) protein sequence database [32] to identify major capsid protein and DNA Pol B candidates. Nucleic acid and amino acid sequences for the major capsid protein (MCP) and DNA Polymerase B (DNA polB) were submitted to Genbank with the accession codes KY509047 and KY509048, respectively.

Phylogenetic analysis was performed using the obtained sequences for MCP and DNA polB, as well as other related sequences from previously discovered algal viruses, identified using BLASTp. These sequences were aligned using the default settings of multiple sequence alignment software version 7 (MAFFT) [33], and trees were constructed from the neighbour-joining method [34] (midpoint-rooted) using Molecular Evolutionary Genetics Analysis version 7.0 (MEGA7) [35].

## 3. Results

### 3.1. Isolation of Lytic Virus Particles

**Figure 1:**
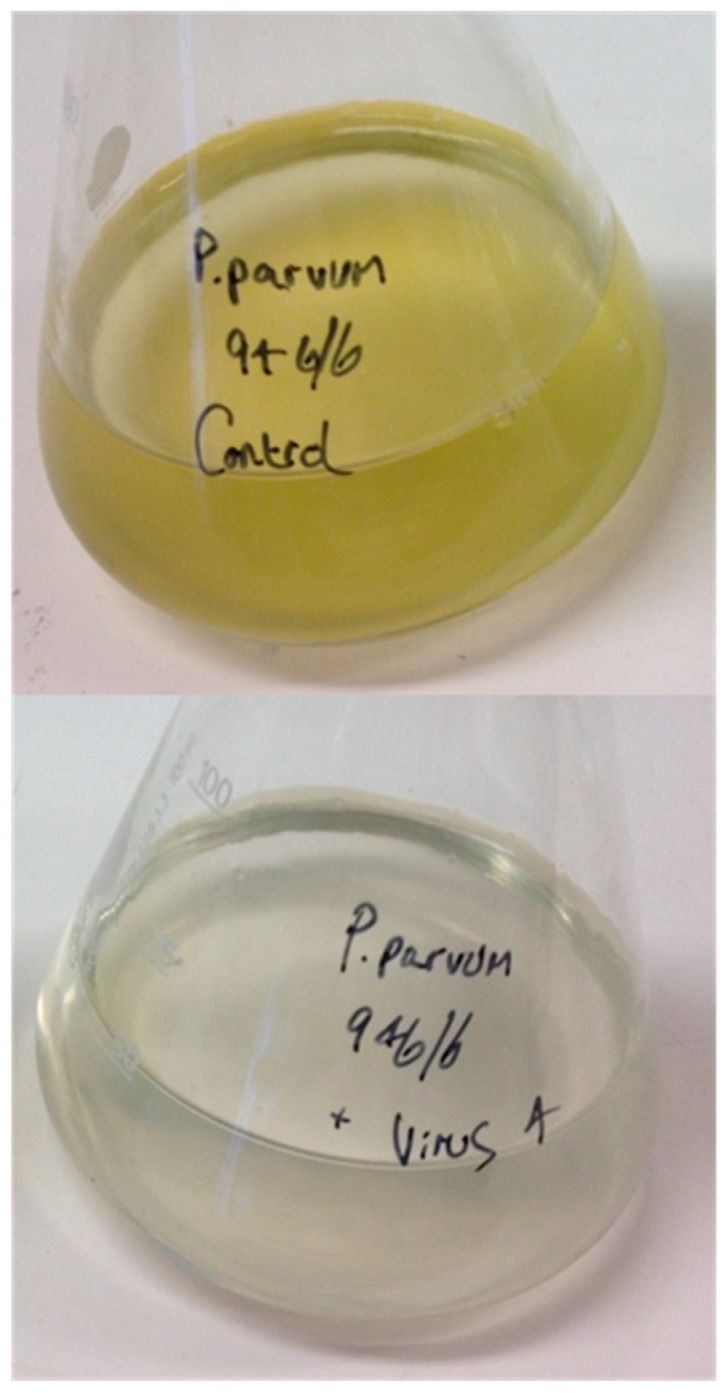
(**Top**) control culture; (**Bottom**) ‘Cleared’ culture 96 h post viral infection.

**Figure 2:**
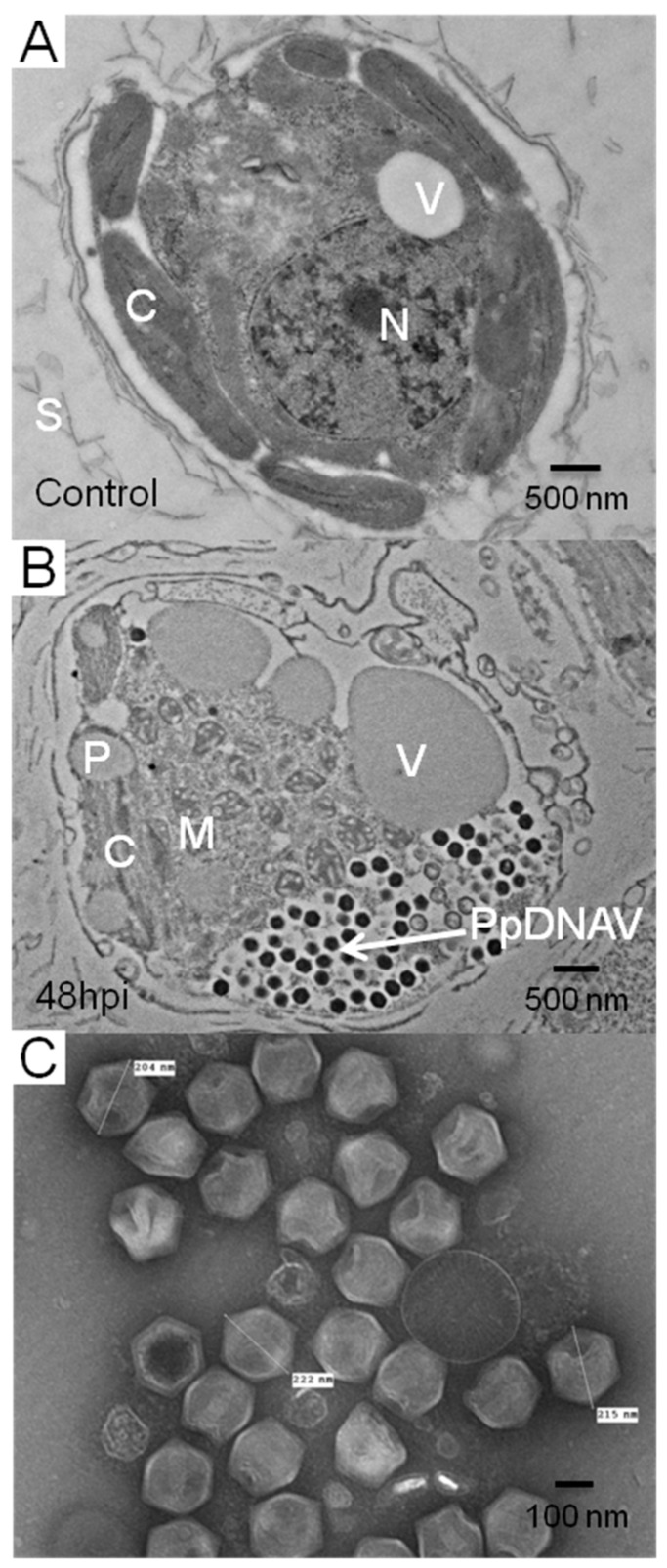
(**A**) Thin-sections of healthy *P. parvum* 946/6 cells; (**B**) Thin-sections of *P. parvum* 946/6 48 h post infection. (**C**) Free *Prymnesium parvum* DNA virus (PpDNAV) particles in culture supernatant 72 h post infection. C: chloroplast; V: contractile vacuole; N: nucleus; S: scales; M: mitochondria, P: pyrenoid.

PpDNAV isolation was conducted from water samples collected at Hickling Broad, Norfolk, England. Among four water samples from which viral lysates were prepared, lysis of *P. parvum* 946/6 occurred with three samples (Figure 1). Transmission electron micrographs of the viral lysates showed that icosahedral VLPs were present in all three samples, but samples 1 and 2 also contained significant levels of phage-like particles; we suspect that these were a result of infection with the low levels of bacteria that were present in the non-axenic *P. parvum* 946/6 cultures. To avoid further downstream separation of viruses, we chose to continue working with sample 4 only (sourced at—52°44′19.12″ N, Long—1°34′39.49″ E), which appeared by TEM to be free of phages. After a triplicate dilution series, the resulting monoclonal viral lysate still lysed the host cells and TEM of thin-sectioned cells confirmed the presence of VLPs (Figure 2A,B); thereby fulfilling Koch’s postulates. 

### 3.2. Virus Morphology, Host Range, and Infectious Properties

Transmission electron microscopy of isolated and intracellular (thin sectioned) viruses revealed an icosahedral capsid with an average diameter of 221 nm (*n* = 71) (Figure 2). Although no external viral lipid membrane was evident, some viral particles showed an internal white ‘halo’ between the capsid and the DNA of PpDNAV, suggestive of the virus having an internal membrane. The presence of a viral factory or viroplasm [36] in the host cytoplasm, and in some cases an imperfect vertex or a tail-like structure were also observed (Figures S1 and S2). As seen in Figure 2B, establishment of a viral factory in the host cytoplasm also results in a loss of the nuclear envelope and therefore loss of the nucleus. This was observed in the majority of infected cells examined at 48 h p.i. 

Fifteen different strains of *Prymnesium* were screened for sensitivity to PpDNAV (Table 1). PpDNAV was found to be sensitive to chloroform, whereby the chloroform-treated virus no longer caused lysis of *P. parvum* 946/6 (Figure S3). This supports the notion of a viral membrane in this system.

**Figure 3:**
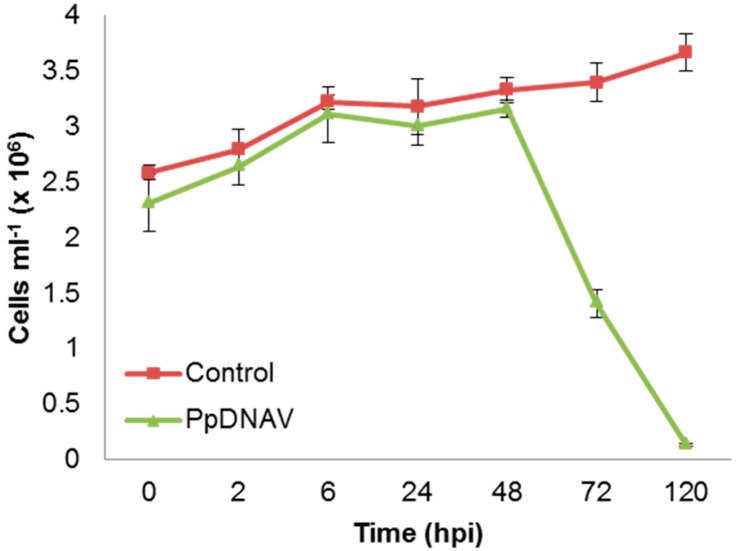
PpDNAV infection cycle propagated on *P. parvum* 946/6. Graph shows the average number of algal cells in control cultures (squares) and PpDNAV infected cultures (circles). Error bars represent the standard error for triplicate cultures.

The lytic cycle of the virus was explored to determine both the incubation period and eclipse period (Figure 3). At 48 h p.i., the cells had clearly lost mobility and sedimented at the base of the culture flask. Re-suspension of the cells by shaking led to similar cell counts as seen at 24 h p.i., as determined by Coulter counting. The time before symptoms of viral infection, the incubation period, was therefore judged to be 24 h. The eclipse period reflects the time between infection and appearance of mature virus particles within the host; as new mature virions were first observed 48 h p.i., the eclipse period was judged to be 24–48 h. At 72 h p.i., the onset of cell lysis had occurred. PpDNAV appeared to lyse >95% of host cells by 120 h p.i., whilst uninfected control cultures continued to grow over the full course of the experiment.

### 3.3. Genome Sequencing and Phylogenetic Analysis

Predicted proteins from the initial genome assembly included the MCP1 protein (KY509047) and DNA polB (KY509048) which were used for phylogenetic analysis. The 525 aa sequence for MCP1 was found to have 91% sequence similarity to the major capsid protein 1 of *Phaeocystis globosa* virus (YP_008052475.1) and 84% similarity to MCP1 of Organic Lake Phycodnavirus 2 (ADX06358.1) with *E*-values of 0.0 in each case. This alignment allowed construction of a phylogenetic tree (Figure S4) that shows clustering with other megaviruses, including PgV-16T.

**Figure 4:**
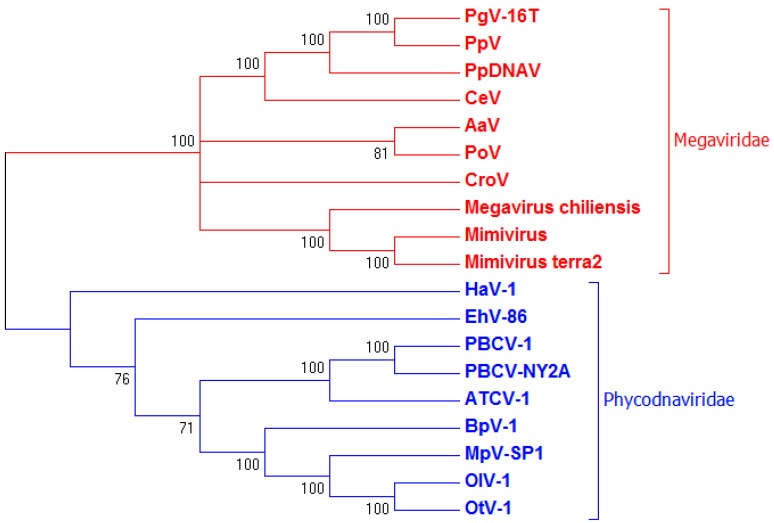
Phylogenetic clustering of PpDNAV with other large algal *Megaviridae*. Alignment was performed using the default settings of multiple sequence alignment software version 7 (MAFFT) [33], and the neighbour-joining method (midpoint-rooted) [34] was used to construct a tree from 19 viral DNA Polymerase Beta (polB) sequences using Molecular Evolutionary Genetics Analysis version 7.0 (MEGA7) [35]. The final tree was based on 630 ungapped positions, 500 resampling permutations, and was collapsed for bootstrap values <50. The tree shows that PpDNAV clusters with the well-defined clade of *Megaviridae* and the algal-infecting *Megaviridae* (red), and not with the *Phycodnaviridae* (blue).

For DNA polB (KY509048), the 1281 aa sequence displayed 77% sequence similarity to DNA polB of PgV-16T (YP_008052566.1) and 64% similarity to DNA polB of Organic Lake phycodnavirus 2 (ADX06483.1). The resulting phylogenetic tree (Figure 4) shows a similar clustering of PpDNAV to the algal *Megaviridae* family, but also illustrates an obvious divergence between algal viruses that fall within the *Megaviridae* family and those that do not; with EhV-86 and *Heterosigma akashiwo* virus (HaV)-1 rightfully placed outside of the *Megaviridae* clade.

## 4. Discussion

Haptophytes are abundant in marine waters but can also thrive in brackish inland waters. Whilst a significant amount of work has been done on the marine dwelling coccolithophore *Emiliania huxleyi* and its associated viruses, little work has looked at the toxin-producing members of the haptophytes. In the present study, we isolated and characterized a novel megavirus, PpDNAV, from brackish inland waters where harmful blooms of *Prymnesium parvum* frequently occur [37]. We showed that this lytic virus was able to infect *P. parvum* 946/6, later expanded to five out of 15 *Prymnesium* strains tested. Morphological and phylogenetic analysis of two core dsDNA virus conserved genes suggests that this virus belongs to the extended *Megaviridae* family of algal-infecting viruses. 

Transmission electron microscopy of negatively stained virus particles from a lysed culture supernatant revealed icosahedral capsids with an average diameter of 221 nm. Many particles appeared to have one imperfect vertex, with some showing material protruding from what appeared to be a stargate [38] (Figure S1). These likely represent particles in an advanced stage of packing or unpacking genetic material [38], and suggested early on that PpDNAV lies in the extended *Megaviridae* branch of algal viruses. Thin sections of infected *P. parvum* 946/6 cells showed evidence for a viroplasm as the site of replication, where empty capsids could be seen closer to the centre of the viroplasm (Figure S2). This further supported the inclusion of PpDNAV in the extended *Megaviridae* family [39]. The infectivity of PpDNAV is chloroform sensitive (Figure S3), and the lack of an obvious external lipid membrane observed by TEM may suggest that internal membrane/s are present; although chloroform sensitivity cannot always be used to confirm lipid membrane presence [40]. 

New mature virions were first observed by electron microscopy at 48 h p.i., so the eclipse period of the virus in infected algal cells was estimated to be 24–48 h. At 48 h, the algal cell count as recorded by Coulter counting was still the same as at 24 h, but a complete sedimentation of cells had occurred, suggesting a loss in motility and a likely shutdown of important cellular processes. By 72 h, a rapid decline in cell abundance could be observed, showing that the loss of motility precedes the host lysis event, as is seen for some other flagellated algae [18]. In its natural environment, this may lead to accumulation of viral particles at the sediment surface rather than dispersed in the water column. 

The algal host species specificity of PpDNAV was assessed against *P. parvum* 946/6, which had been kept in 7–8 PSU f/2 medium for two years, and 14 other *Prymnesium* strains which had been maintained in a full strength seawater medium. Initially, PpDNAV only infected *P. parvum* 946/6, but after ~6 months of sub-culturing of the other 14 strains in 7–8 PSU f/2 medium, the host range broadened to five out of the 15 strains. We speculate that the change in salinity contributed to the change in sensitivity to PpDNAV; recent work by Nedbalová et al. [41] suggests that a change in membrane lipid composition in different salinities may account for this situation. This somewhat less restricted host range is similar to that found for *Haptolina ericina* virus (HeV RF02), *Prymnesium kappa* virus (PkV RF01) and *Prymnesium kappa* virus (PkV RF02) [42]. Taken together, this suggested that PpDNAV was a member of the algal *Megavirus* family [43].

Phylogenetic analysis using sequences for MCP1 and DNA polB of PpDNAV confirmed morphological findings, showing that PpDNAV clusters amongst the algal viruses belonging to the *Megaviridae* family, such as PgV-16T [3,6], *Chrysochromulina ericina* virus (CeV) [44], *Pyramimonas orientalis* virus (PoV) [45] and the recently reassigned *Aureococcus anophagefferens* virus (AaV) [46]. With the exception of *Emliania huxleyi* virus (EhV-86), which appears to branch independently, the close clustering of viruses infecting haptophytes, as well as the chlorella viruses clustering together, supports the notion that viruses co-evolve with their hosts [6,46,47].

Of the algal viruses compared in this study, only HaV-1 is known to infect a toxin producing host [12,13,14]. However, the toxic metabolites responsible for bloom toxicity are not established in *Heterosigma akashiwo*, making studies of viral impact on toxicity difficult. On the other hand, reports of toxic *P. parvum* metabolites are numerous and include fatty acids [48], glycerolipids [49] and very large ladder-frame polyether toxins, known collectively as the prymnesins [50,51,52]. Reports of cases of toxic and non-toxic blooms of *Prymnesium* and other harmful algal species [37] has led to speculation that an ecological trigger exists for toxicity. While efforts have been made to associate nutrients, pH and other conditions to bloom toxicity [21], the identity of the full spectrum of toxicity-causing agents remains to be establshed; there may conceivably be a role for viral infection in *Prymnesium* cell lysis and hence toxin release. We now have the opportunity to use this algae–virus system in clearing up some of these unanswered questions. Further studies into the effect of viral infection and host algal cell lysis on toxic bloom events need to be explored in order to fully understand the underlying mechanisms behind production and release of toxins from Prymnesium. In addition, as further sequences of algal viruses become available, new opportunities will open up for accurate monitoring of viral population fluctuations with respect their host. Furthermore, the increase in characterized viruses will provide more information when analysing metagenomic data sets such as those generated by the *Tara* Oceans expedition [53,54]. Hence the discovery and characterization of PpDNAV in this study will aid this burgeoning field of scientific endeavour.

## Figures and Tables

**Table 1:** Host range of PpDNAV. + lysed culture, − culture not lysed.

Genus/Species	Strain Code	Lysis with PpDNAV
*Prymnesium parvum*	946/6	+
*Prymnesium parvum*	94A	-
*Prymnesium parvum*	94C	+
*Prymnesium parvum*	579	-
*Prymnesium patelliferum*	527A	+
*Prymnesium patelliferum*	527C	+
*Prymnesium patelliferum*	527D	-
*Prymnesium sp.*	522	-
*Prymnesium sp.*	569	-
*Prymnesium sp.*	592	+
*Prymnesium sp.*	593	-
*Prymnesium sp.*	595	-
*Prymnesium sp.*	596	-
*Prymnesium sp.*	597	-
*Prymnesium sp.*	598	-

## Supplementary Materials

The following are available online at www.mdpi.com/1999-4915/9/3/40/s1. Figure S1: Electron micrographs showing PpDNAV particles with an imperfect vertex; Figure S2: Electron micrographs showing both mature and immature virions close to a viroplasm; Figure S3: Chloroform sensitivity as judged by culture clearing; Figure S4: Neighbor-joining phylogenetic tree of MCPs.
