# Supplementary material for: Isolation and Characterization of a Double Stranded DNA Megavirus Infecting the Toxin-Producing Haptophyte Prymnesium parvum
Source: Viruses. 2017 Mar 9;9(3):40. doi: 10.3390/v9030040 (PMC5371795; doi:10.3390/v9030040)
Supplement: Supplementary file 1 [file SI Figure 4.pdf › supplementary revised/SI Figure 4.pdf]

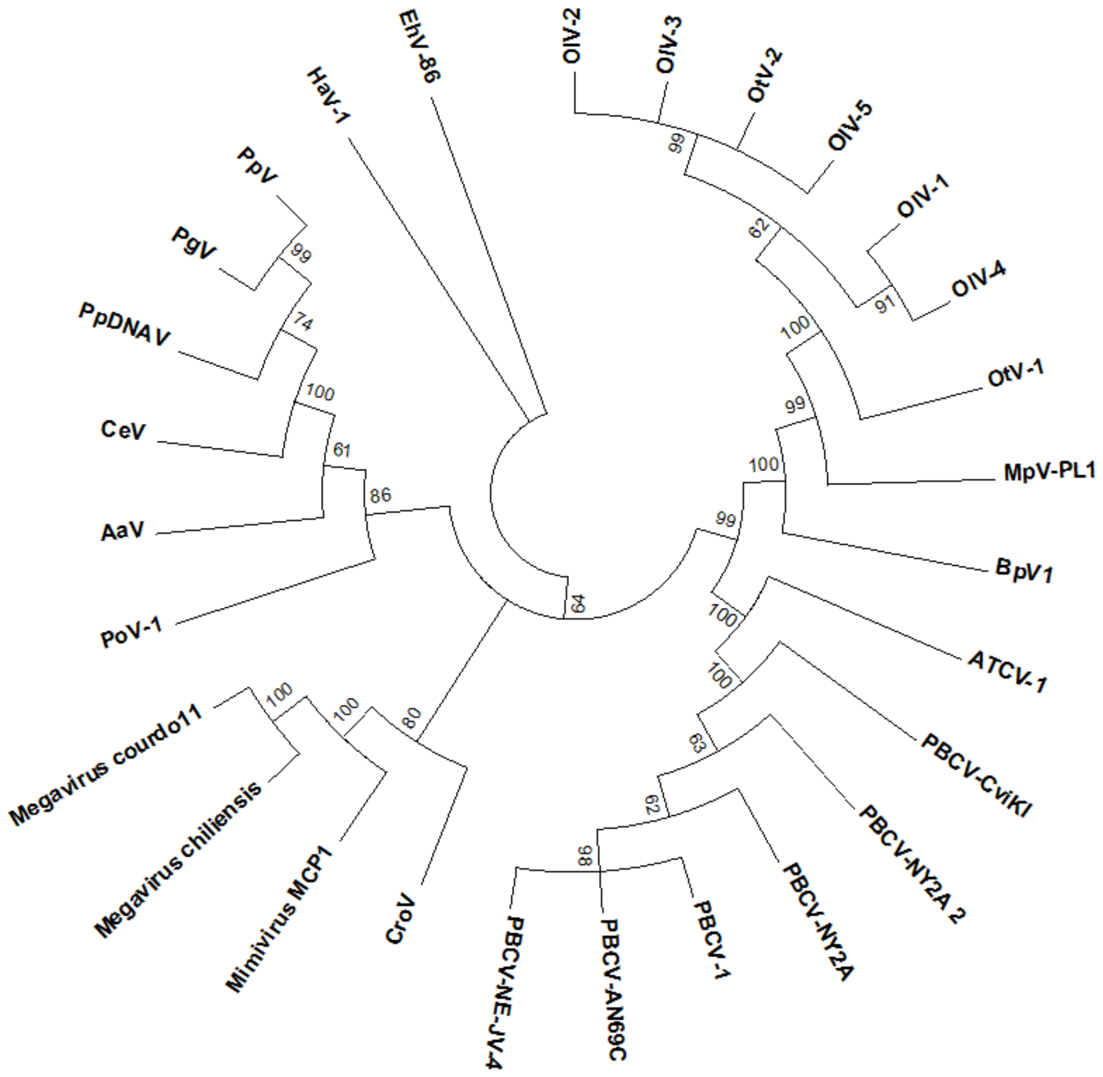

SI Figure 4. Phylogenetic clustering of PpDNAV with other large algal *Megaviridae*. Alignment was performed using the default settings of MAFFT [1], and the Neighbour-joining method (midpoint-rooted) [2] was used to construct a tree from 28 viral MCP sequences using MEGA7 [6]. The final tree was based on 336 ungapped positions, 500 resampling permutations, and was collapsed for bootstrap values <50. The tree shows that PpDNAV clusters with the algal-infecting *Megaviridae*, separate to the *Phycodnaviridae*.
