# Supplementary material for: Isolation and Characterization of a Double Stranded DNA Megavirus Infecting the Toxin-Producing Haptophyte Prymnesium parvum
Source: Viruses. 2017 Mar 9;9(3):40. doi: 10.3390/v9030040 (PMC5371795; doi:10.3390/v9030040)
Supplement: Supplementary file 1 [file viruses-09-00040-s001.zip › supplementary revised/SI Figure 1.pdf]

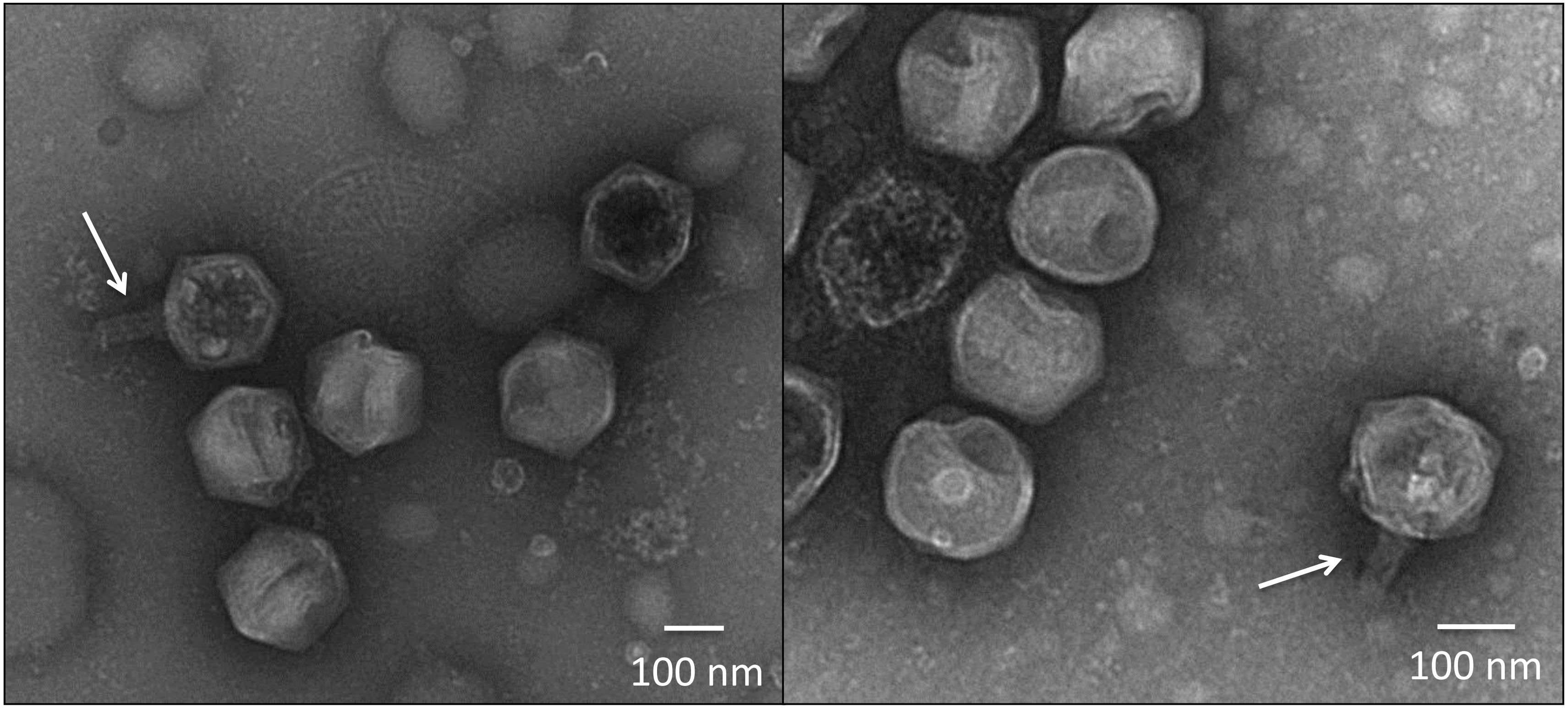


**SI Figure 1.** Electron micrographs of negatively stained PpDNAV particles. A small number of particles appear to have a single imperfect vertex, likely representing a stargate structure (white arrow).
