# Supplementary material for: Isolation and Characterization of a Double Stranded DNA Megavirus Infecting the Toxin-Producing Haptophyte Prymnesium parvum
Source: Viruses. 2017 Mar 9;9(3):40. doi: 10.3390/v9030040 (PMC5371795; doi:10.3390/v9030040)
Supplement: Supplementary file 1 [file viruses-09-00040-s001.zip › supplementary revised/SI Figure 2.pdf]

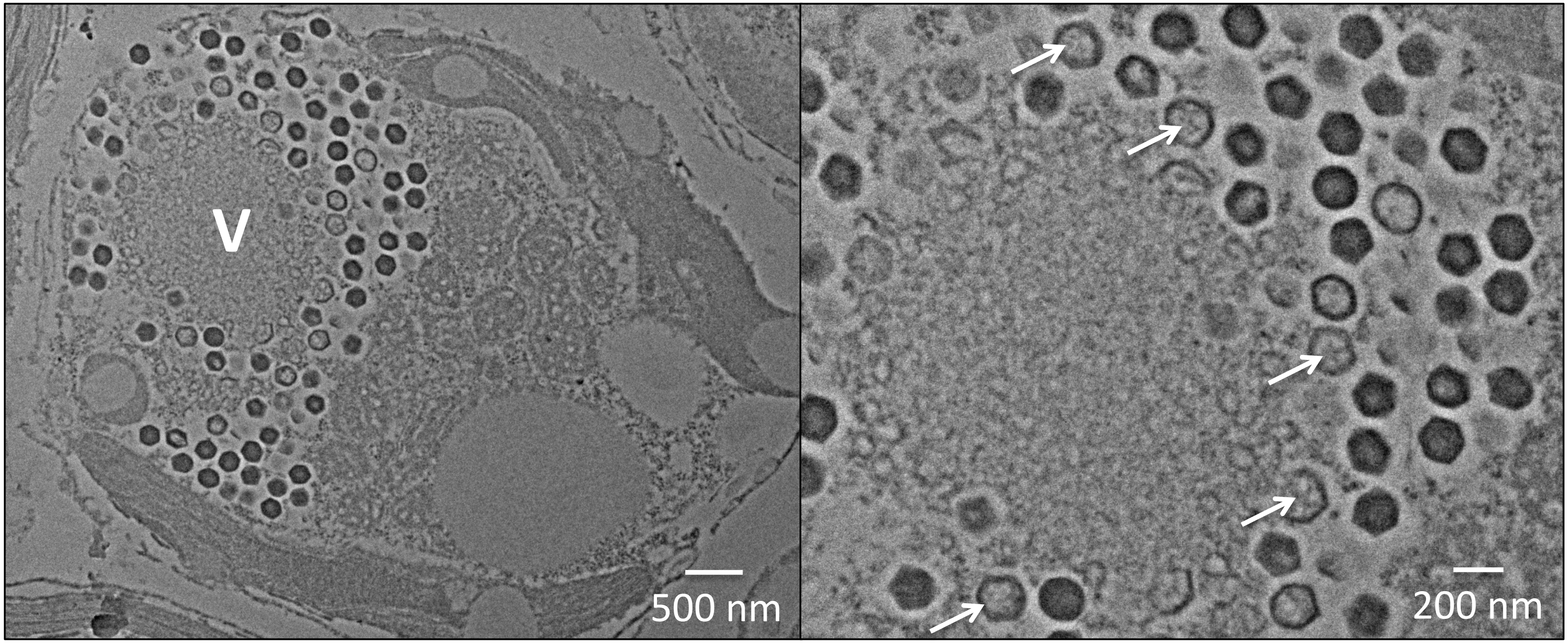

SI Figure 2. Electron micrographs of infected *P. parvum* 946/6 48 hpi. Left – Whole cell visually infected with many PpDNAV particles surrounding a viroplasm. Right – Magnified from left. Both mature and immature particles can be observed. ‘Empty’ particles are denoted by a white arrow. V – viroplasm.
