# Supplementary material for: Isolation and Characterization of a Double Stranded DNA Megavirus Infecting the Toxin-Producing Haptophyte Prymnesium parvum
Source: Viruses. 2017 Mar 9;9(3):40. doi: 10.3390/v9030040 (PMC5371795; doi:10.3390/v9030040)
Supplement: Supplementary file 1 [file viruses-09-00040-s001.zip › supplementary revised/SI Figure 3.pdf]

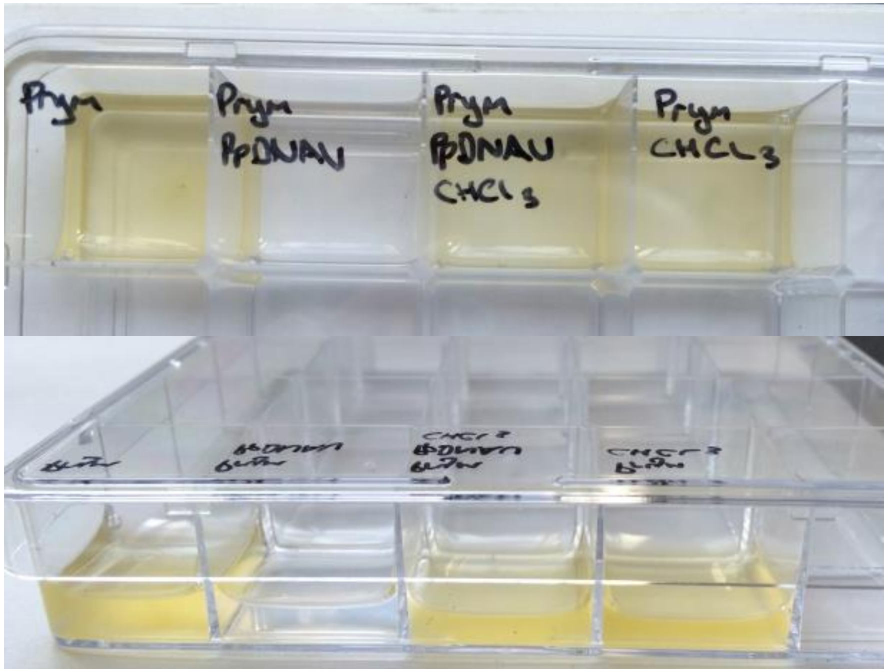

SI Figure 3. Chloroform sensitivity assay. Left to right – *P. parvum* 946/6 + f/2 media; *P. parvum* 946/6 + PpDNAV; *P. parvum* 946/6 + Chloroform treated PpDNAV; *P. parvum* 946/6 + Chloroform treated f/2 media.
